# Neuron-Derived Sema3B Facilitates Microglial Hematoma Clearance After Intracerebral Hemorrhage

**DOI:** 10.3390/antiox15020220

**Published:** 2026-02-08

**Authors:** Baisong Huang, Anqi Chen, Tong Zhou, Ying Xu, Yuanyuan Sun, Quanwei He

**Affiliations:** Department of Neurology, Union Hospital, Tongji Medical College, Huazhong University of Science and Technology, 1277 Jiefang Avenue, Wuhan 430022, China

**Keywords:** microglia, hematoma clearance, semaphorin class 3B, Nrf2, HO-1, Trem2

## Abstract

Intracerebral hemorrhage (ICH) is the deadliest subtype of stroke, and its primary harm to the human body arises from the formation of brain hematomas. Promoting microglial-mediated endogenous hematoma clearance has become a key focus in current ICH treatment strategies. Semaphorin 3s (Sema3s) are molecular signals involved in the regulation of the central nervous system, angiogenesis, and microenvironment homeostasis, and they are closely associated with various central nervous system diseases. Hematoma clearance and inflammation regulation are crucial to the role of microglia, yet the mechanisms by which Sema3s regulate microglia after ICH remain unclear. Here, using high-throughput RNA sequencing of a mouse ICH model, we identified that neuron-derived Sema3B is downregulated after ICH. Further mechanistic studies revealed that Sema3B can bind to PlexinA1 on microglia, activating NRF2 to promote the expression of the phagocytic receptor TREM2 and the key hematoma clearance molecule HO-1. Furthermore, Sema3B enhances the interaction between PlexinA1 and TREM2, cooperatively boosting microglial phagocytosis of the hematoma after ICH. Furthermore, Sema3B regulates the M2 polarization of microglia, exerting an anti-inflammatory effect. Our findings suggest that manipulating microglial phagocytosis of hematoma and inflammation suppression via regulation of Sema3B may be a potential strategy for treating patients with ICH.

## 1. Introduction

According to the latest data from the Global Burden of Disease (GBD) study, stroke continues to be the second leading cause of death globally, contributing significantly to the disease burden and societal expenses [1]. Against this backdrop, although intracerebral hemorrhage (ICH) accounts for only 10–20% of stroke cases, it is the subtype with the poorest prognosis; its striking short-term mortality (up to 30–40%) renders it one of the most urgent clinical challenges in neuroscience [2]. Spontaneous ICH is mainly caused by the sudden and unexpected breaking of blood vessels in the brain, often accompanied by factors such as high blood pressure, abnormal blood vessel formations, clotting disorders, and the transformation of ischemic stroke into a hemorrhagic state [3]. The predominant source of injury in ICH is the formation of the intraparenchymal hematoma which constitutes the primary injury. The hematoma can rapidly elevate intracranial pressure, thereby precipitating cerebral hypoperfusion, brain herniation, and even fatal outcomes. In the subsequent secondary injury phase, lysis of erythrocytes within the hematoma releases hemoglobin, heme, and free iron, which, through oxidative stress and excessive inflammatory responses, lead to cerebral edema, excitotoxicity, and neuronal death [2]. The combined effects of these primary and secondary injury mechanisms drive the rapid clinical deterioration observed in ICH; therefore, the volume of the hematoma is widely regarded as the most critical predictor of mortality outcome [4]. Early hematoma clearance is therefore crucial for improving outcomes. At present, surgical evacuation remains the principal approach for removing ICH hematomas; however, surgical intervention has not been conclusively shown to confer substantial improvements in functional outcome or mortality, and it carries risks of invasiveness, additional tissue injury, and infection [5]. Moreover, many patients with ICH are not suitable candidates for surgery (e.g., those with severe diabetes or hypertension and smaller-volume hemorrhages) [6]. Therefore, the exploration and development of therapeutic strategies that promote endogenous hematoma clearance while mitigating brain injury and restoring neurological function constitute the current priority in ICH research.

Microglia, which serve as the primary professional phagocytes in the central nervous system (CNS), play a critical role in clearing hematomas after ICH. They achieve this by engulfing components of the hematoma, including erythrocytes, hemoglobin, and heme [7]. Augmenting microglial erythrophagocytosis before erythrocyte lysis can ameliorate neurological deficits in ICH mice, attenuate perihematomal inflammation, and thereby limit brain injury [7]. Following ICH, perihematomal microglia polarize toward a proinflammatory M1 phenotype and an M2 phenotype that suppresses inflammation and promotes phagocytosis. M2-polarized microglia offer multifaceted protective benefits against secondary brain injury caused by ICH by enhancing the activity of nuclear factor erythroid 2–related factor 2 (Nrf2) at the gene expression level, a crucial regulator involved in hematoma clearance following ICH [8,9]. Nrf2 promotes the engulfment of hematoma debris by microglia through the upregulation of proteins associated with phagocytosis and also influences the synthesis of heme oxygenase-1 (HO-1). HO-1, an enzyme essential for controlling the breakdown of heme, accelerates the removal of neurotoxic heme by microglia, helping to alleviate oxidative stress and neuroinflammation after ICH [8]. Additionally, recent findings suggest that Nrf2 plays a direct role in modulating the expression of triggering receptor expressed on myeloid cells 2 (TREM2) in microglia [9].

Activation of TREM2 is indispensable for the capacity of microglia to counter oxidative stress and mitigate neuroinflammation after ICH. TREM2, a critical innate immune receptor primarily found on brain microglia, is involved in regulating microglial survival, proliferation, phagocytic activity, and cytokine secretion [10]. Within the field of neurodegenerative and cerebrovascular disorders, including Parkinson’s disease, Alzheimer’s disease, and IS, TREM2 is widely recognized for its role in mediating neuroprotective functions [11]. In recent years, TREM2 has also been shown to confer neuroprotection in ICH, often via the phosphatidylinositol 3-kinase/protein kinase B (PI3K/Akt) pathway. Experimental studies in mouse ICH models indicate that TREM2 activation via this pathway promotes the recovery of neurological capabilities, diminishes inflammatory damage and programmed cell death in neurons [12], but also attenuates white matter injury after ICH [13]. Additionally, increasing the expression of TREM2 in the brains of mice helps to improve neurological impairments caused by ICH, while also mitigating neuroinflammation, decreasing cell death, and alleviating swelling in the brain [14]. Notably, emerging evidence indicates that TREM2 function is not exerted in isolation; it may form complexes with other receptors to receive extracellular signals. One study demonstrated a direct protein–protein interaction between TREM2 and PlexinA1 [15], a member of the plexin family, suggesting that PlexinA1 may act as a critical regulatory partner of TREM2, participating in its signal transduction or functional modulation. Importantly, the functional interplay between these receptors may underlie critical neuron-microglia communication, as neurons are known to participate in modulating microglial phagocytosis post-ICH.

Notably, PlexinA1 is a principal receptor for class 3 semaphorins (Semaphorin 3s, Sema3s). Class 3 semaphorins comprise a group of secreted proteins comprising seven members (Sema3A–Sema3G) that bind, in autocrine and paracrine fashions, to PlexinA1–A4 receptors on the cell surface to mediate downstream signal transduction, intercellular communication, and effector functions [16,17]. Recent studies have implicated Sema3s as molecular cues in neural modulation, angiogenesis, and microenvironmental homeostasis within the CNS, with close associations to multiple CNS disorders [18]. For example, Sema3A regulates the permeability of cerebrovascular endothelial cells and exacerbates brain injury after IS [19]; Sema3C has been demonstrated to direct and facilitate axonal outgrowth in dopaminergic neurons, indicating its promising therapeutic applications in Parkinson’s disease [20]; meanwhile, Sema3D has been identified as a genetic susceptibility locus associated with schizophrenia and is also recognized as an emerging risk factor linked to age-related cognitive decline [21,22]. Sema3B is expressed predominantly in neurons [18]; in the CNS, Sema3B interacts with its primary receptor, PlexinA1, playing a crucial role in directing the growth and positioning of axonal projections across the ventral midline during the developmental stages of vertebrate embryos [23,24,25]. In recent years, transcriptomic data from patients and animal models of post-traumatic stress disorder (PTSD) have implicated Sema3B in PTSD [26], and Sema3B has also been reported as a regulator of synaptic plasticity and neuronal survival, contributing to the pathogenesis of depression [27]. Sema3B can further promote appropriate recognition and ensheathment between unmyelinated axons and Schwann glia, thereby regulating axon–axon and axon–glia interactions, with the potential to accelerate recovery from peripheral nerve injury and alleviate associated pain [28]. Despite its recognized roles in neural development, the contribution of Sema3B/PlexinA1 signaling to the pathophysiology of ICH is poorly defined. The present work was designed to test the premise that neuron-derived Sema3B binds to its receptor PlexinA1 on microglia and activating NRF2 to promote the expression of the phagocytic receptor TREM2 and the key hematoma clearance molecule HO-1. Furthermore, an interaction exists between PlexinA1 and TREM2, which is enhanced by Sema3B. This enhancement significantly amplifies the TREM2-mediated phagocytic pathway, thereby promoting more effective hematoma clearance. Furthermore, Sema3B regulates the M2 polarization of microglia, exerting an anti-inflammatory effect. Our findings suggest that manipulating microglial phagocytosis of hematoma and inflammation suppression via regulation of Sema3B may be a potential strategy for treating patients with ICH.

## 2. Methods and Materials

### 2.1. ICH Model and Animals

The Animal Ethics Committee of Tongji Medical College, Huazhong University of Science and Technology, reviewed and approved all procedures involving mice. In this study, we utilized healthy adult male C57BL/6 mice (8 weeks old; 22–25 g) obtained from Hubei Beiante Biotechnology Co., Ltd., Wuhan, China. These animals were acclimatized and maintained under standardized specific pathogen-free (SPF) conditions, with continuous access to a standard laboratory diet and water throughout the study period. All animals were acclimatized for at least one week prior to the induction of any experimental models. Mice that died due to anesthesia or failure of model establishment were excluded. We induced ICH in mice through stereotaxic administration of collagenase type IV (Med Chem Express, Shanghai, China) under sterile conditions. Animals were anesthetized with 2% isoflurane throughout the surgery. After ensuring a stable depth of anesthesia, each mouse was positioned in a stereotaxic instrument for precise cranial fixation. The scalp hair was removed, and the skin was disinfected alternately with povidone–iodine and 75% ethanol. A midline incision approximately 1 cm in length was made to clearly reveal the bregma. Guided by a standard mouse brain atlas, the right striatum was localized for injection. The stereotaxic coordinates were set relative to bregma at +0.2 mm anteroposterior, the midline at +2.0 mm mediolateral (right hemisphere), and the skull surface at −3.3 mm dorsoventral. Meticulous drilling with a micro-drill was used to create a small cranial window at the target location, with the dura mater left intact. A 1 μL microsyringe (Hamilton, NV, USA) was loaded with 0.08 U of type IV collagenase in 0.8 μL of sterile saline and securely attached to the stereotaxic arm. The needle was gradually advanced to the target depth, and the collagenase solution was administered at a constant rate over a period of 5 min. Following the infusion, the needle was left in place for 5 min to allow the solution to diffuse. This pause was intended to prevent reflux before the needle was withdrawn slowly.

### 2.2. Intracerebroventricular Injection of Recombinant Sema3B and AAV-shPlexinA1

Mice were anesthetized with 1–2% isoflurane for induction and maintenance. After the mice reached a deep level of anesthesia, intracerebroventricular injections were performed using a stereotaxic apparatus (Ruiwode Life Science, Shenzhen, China). Recombinant mouse Sema3B protein (R&D Systems, Minneapolis, MN, USA) was administered via intracerebroventricular injection at the following coordinates: AP −0.3 mm, ML +1.0 mm, DV −2.5 mm. The total dose of Sema3B was 300 ng dissolved in 3 μL, delivered at an infusion rate of 0.1 μL/min. Mice in the Sema3B treatment group received recombinant Sema3B prior to ICH induction. After injection, the needle was left in place for 5 min to prevent reflux, then slowly withdrawn, and the scalp incision was sutured.

AAV-shPlexinA1 and the corresponding negative control AAV were purchased from GeneChem Co., Shanghai, China. The interference AAV vector contained the Iba1p-EGFP-mir155 (mcs)-SV40 PolyA cassette. Mice in the AAV-shPlexinA1 treatment group received viral injection 1 month prior to ICH induction. Under 1–2% isoflurane anesthesia, mice underwent intracerebroventricular injection using a stereotaxic apparatus at the same coordinates (AP −0.3 mm, ML +1.0 mm, DV −2.5 mm). The total viral dose was 1 × 10^10^ vg, with a titer of 1 × 10^13^ vg/mL, delivered in a volume of 1 μL at an infusion rate of 0.1 μL/min. After injection, the needle was retained for 5 min to prevent reflux, then slowly withdrawn, and the scalp incision was sutured.

### 2.3. Perihematomal Tissue Collection

Perihematomal tissue was collected at the indicated time points after ICH. Mice were euthanized and transcardially perfused with PBS to minimize intravascular blood contamination. Brains were rapidly removed and sectioned into coronal slices using a mouse brain matrix (Servicebio, Wuhan, China). The hematoma core was visually identified and carefully removed. Perihematomal tissue was then microdissected as the region within approximately 1 mm surrounding the hematoma margin and used for downstream assays. For sham controls, tissue was collected from the anatomically matched region using the same sectioning and dissection strategy.

### 2.4. RNA Sequencing

Perihematomal tissue was harvested from mice on day 3 after ICH, and tissue from the corresponding brain region was collected from sham-operated mice. Total RNA was extracted using TRIzol (Invitrogen, 15596026, Waltham, MA, USA) according to the manufacturer’s instructions, and RNA integrity was assessed using an Agilent 2100 Bioanalyzer. Transcriptome libraries were prepared using the Singleron bulk-RNA library preparation workflow and sequenced on the Illumina NovaSeq 6000 platform. Differential expression analysis was performed with DESeq2, and differentially expressed genes were defined as those with padj ≤ 0.05 and |fold change| ≥ 1.5. Volcano plots were generated using R (v4.3.2).

### 2.5. Neurological Deficit Score (NDS)

Neurological function was evaluated 3 days after ICH induction in a quiet environment by two investigators blinded to group allocation. A modified 28-point NDS was applied, including assessments of Gait, Body symmetry, Front limb symmetry, Circling behavior, Climbing, Whisker response, and Compulsory circling. Final results were presented as the mean score for each mouse.

### 2.6. Hematoma Volume Detection

To quantify the hematoma volume, mice were euthanized on day 3 post-ICH induction via collagenase injection. Following transcardial perfusion, the brains were harvested and immersion-fixed in 4% PFA for 24 h. Serial 1 mm thick coronal sections were then prepared. An investigator blinded to the experimental groups delineated the hematoma margins in five consecutive sections using image analysis software (Image-Pro Plus 6.0). The total volume was calculated by summing the areas of hemorrhage from these sections and multiplying by the intersection distance (1 mm).

### 2.7. Hemoglobin Content Measurement

To assess the extent of hemorrhage, mice were transcardially perfused 72 h after ICH induction. The ipsilateral hemisphere was dissected, homogenized in ice-cold PBS, and centrifuged (15,000 g, 30 min, 4 °C). The resulting supernatant was analyzed for hemoglobin content using a commercial assay kit (Beyotime Biotech, Shanghai, China), following the manufacturer’s protocol. The absorbance was measured at 410 nm for quantification.

### 2.8. Cell Culture and Treatment

Mouse microglial BV2 cells and mouse neuroblastoma N2a cells were obtained from LiScien Biotechnology (Shenzhen, China) and grown in DMEM medium (Servicebio, Hubei, China) containing 10% fetal bovine serum (Sciencell, Carlsbad, CA, USA), 1% streptomycin, and 1% penicillin (Servicebio, China). Cells were exposed to 10 μM hemoglobin (Hb; Solarbio, Beijing, China) for 24 h. This treatment was utilized to model ICH conditions in an in vitro setting. For Sema3B treatment, recombinant Sema3B (R&D Systems, USA) was added at 200 ng/mL 1 h prior to Hb induction. For Syk kinase inhibition, R406 (MCE, Monmouth Junction, NJ, USA) was added at 5 μM 12 h before Sema3B treatment. Western blotting and immunofluorescence staining were utilized to determine changes in protein levels at the 12 h time point following Hb exposure.

### 2.9. siRNA Transfection

BV2 cells at 40% confluence were transfected via a 48 h incubation with the appropriate transfection reagent (D-Nano Therapeutics, Beijing, China), together with siRNAs targeting TREM2 (forward 5′-CCGUCACCAUCACUCUGAAGAUU-3′; reverse 5′-UUCAGAGUGAUGGUGACGGUUUU-3′), PlexinA1 (forward 5′-GGUAGUAUACGAACUGCUCGC-3′; reverse 5′-GCGAGCAGUUCGUAUACUACC-3′), and Nrf2 (forward 5′-CGACAGAAACCUCCAUCUACU-3′; reverse 5′-AGUAGAUGGAGGUUUCUGUCG-3′), or a negative control (NC) siRNA (forward 5′-UUCUCCGAACGUGUCACGU-3′; reverse 5′-ACGUGACACGUUCGGAGAA-3′). Transfection efficiency was verified by Western blotting, after which subsequent experiments were performed.

### 2.10. Erythrophagocytosis Assay

Using gradient centrifugation, red blood cells (RBCs) were isolated from mouse peripheral blood and washed three times with PBS. The RBCs were incubated with 1,1′-Dioctadecyl-3,3,3′,3′-Tetramethylindodicarbocyanine, 4-Chlorobenzenesulfonate Salt (DiD; a lipophilic cell membrane fluorescent probe; Beyotime Biotech, Wuhan, China) at 37 °C for 20 min, and then washed three times with PBS to generate DiD-labeled RBCs. Subsequently, DiD-labeled RBCs were co-incubated with BV2 microglial cells that had been cultured in serum-free medium for 12 h at 37 °C for 4 h in the dark. The ratio of RBCs to BV2 microglial cells was 10:1. After incubation, RBC lysis buffer (Solarbio, Beijing, China) was used to remove RBCs that had not been phagocytosed by BV2 microglial cells, and the cells were washed three times with PBS, leaving BV2 microglial cells. A flow cytometer (BD, Franklin Lakes, NJ, USA) was used to detect their fluorescence signals, and the mean fluorescence intensity was analyzed.

Meanwhile, microglial cells seeded on coverslips were subjected to the same DiD staining procedures as described above. After staining, the cells were fixed with 4% paraformaldehyde (Servicebio, Wuhan, China). After fixation, the cells were washed three times with PBS, and then blocked for 30 min with 10% donkey serum containing 0.1% Triton X-100. The cells were incubated with an Iba-1 antibody (1:1000, Abcam, Waltham, MA, USA) at 4 °C overnight. After washing the samples three times with PBS, the samples were incubated with the corresponding fluorophore-conjugated antibody (1:200, Invitrogen, USA) at room temperature for 1 h. After washing three times with PBS, DAPI (Beyotime Biotech, Wuhan, China) was used to stain the nuclei for 10 min. Finally, a confocal microscope (Nikon, Tokyo, Japan) was used to observe their fluorescence intensity.

### 2.11. Flow Cytometry

Harvested BV2 cells were prepared in 220 μL of PBS for immediate analysis on a flow cytometer (BD, USA). The aim was to detect and quantify the Iba1-positive microglia that exhibited DiD fluorescence, indicating erythrocyte uptake.

### 2.12. Western Blot and Co-IP

Protein was extracted from cell and animal tissue samples using a protein lysis buffer (Servicebio, China) supplemented with a protease inhibitor cocktail (MCE, China). The protein concentration was determined using a BCA assay kit (Beyotime Biotech, China). Equal amounts of protein were separated by gel electrophoresis and then transferred onto PVDF membranes (Millipore, Burlington, MA, USA). The membranes were blocked at room temperature for one hour with 5% non-fat milk in TBST, followed by an overnight incubation with primary antibodies. The antibodies used are as follows: Sema3B (1:2000, R&D Systems, USA), PlexinA1 (1:2000, Abcam, USA), Nrf2 (1:2000, Proteintech, Wuhan, China), TREM2 (1:2000, Proteintech, China), HO-1 (1:2000, Proteintech, China), PI3K (1:2000, Proteintech, China), phospho-PI3K (1:2000, Proteintech, China), AKT (1:2000, Proteintech, China), phospho-AKT (1:2000, Proteintech, China), SYK (1:2000, Proteintech, China), phospho-SYK (1:2000, Proteintech, China), mTOR (1:2000, Proteintech, China), phospho-mTOR (1:2000, Proteintech, China), p65 (1:2000, Proteintech, China), phospho-p65 (1:2000, Proteintech, China), IκB-α (1:2000, Proteintech, China), phospho-IκB-α (1:2000, Proteintech, China), CD206 (1:2000, Proteintech, China), CD86 (1:2000, Abclonal, China), DAP12 (1:2000, CST, Danvers, MA, USA), and GAPDH (1:3000, Proteintech, China). Subsequent to washing, a 2 h room temperature incubation with HRP-linked secondary antibodies (1:5000; ABclonal Technology, Wuhan, Hubei, China) was performed. Signal development was achieved with an ECL system, followed by image acquisition and quantitative analysis of the bands with ImageJ (v1.54).

We used an anti-PlexinA1 antibody (Abcam, USA) conjugated to agarose magnetic beads (MCE, China) to pull down interacting proteins from lysates of BV2 cells (2 × 10^7^). The immunoprecipitated complexes were then resolved and detected by Western blotting according to the established method.

### 2.13. Quantitative Real-Time PCR

Total RNA was extracted from fresh tissue and cell samples using a specified kit (CINOTOHI, Changsha, Hunan, China). The extracted RNA was then reverse-transcribed into cDNA using a commercial reagent (HYcezmbio, Wuhan, Hubei, China). Quantitative real-time PCR analysis was conducted on an Invitrogen real-time PCR system using SYBR Green Master Mix (HYcezmbio, China), and GAPDH was employed as an internal control for normalization. The following primers were used: Sema3B: forward 5′-GAGGACTCTGCCGCTATCAC-3′, reverse 5′-CTCCACACCCAACACCTTCT-3′; PlexinA1: forward 5′-ACCCTGTACTGGAGCCACTTAGC-3′, reverse 5′-GCCGATGAGCACCGTGTAGTTG-3′; TREM2: forward 5′-GAAGAAGCGGAATGGGAGCACAG-3′, reverse 5′-CCTCGGAGACTCTGACACTGGTAG-3′; Nrf2: forward 5′-CTTTAGTCAGCGACAGAAGGAC-3′, reverse 5′-AGGCATCTTGTTTGGGAATGTG-3′; IL-1β: forward 5′-GAAATGCCACCTTTTGACAGTG-3′, reverse 5′-TGGATGCTCTCATCAGGACAG-3′; IL-6: forward 5′-TGGGGCTCTTCAAAAGCTCC-3′, reverse 5′-AGGAACTATCACCGGATCTTCAA-3′; TNF-α: forward 5′-GCGGCCACAGAAAACACTC-3′, reverse 5′-CTCCCAATGGTCAAGGCATC-3′; GAPDH: forward 5′-AATGGATTTGGACGCATTGGT-3′, reverse 5′-TTTGCACTGGTACGTGTTGAT-3′; HO-1: forward 5′-ACCGCCTTCCTGCTCAAC ATTG-3′, reverse 5′-CTCTGACGA AGTGACGCCATCTG-3′.

### 2.14. Enzyme-Linked Immunosorbent Assay (ELISA)

We used an ELISA kit (Biobyt, Cambridge, UK) to measure Sema3B in the peri-hematomal brain tissue of mice. We quantified the levels of interleukin-1β (IL-1β), interleukin-6 (IL-6), and tumor necrosis factor-α (TNF-α) in mouse perihematomal brain tissue homogenates. Measurements were performed with standard colorimetric ELISA kits procured from Boster (Wuhan, Hubei, China).

### 2.15. Immunofluorescence Staining

Following blockage with 10% donkey serum/0.1% Triton X-100, sections were probed overnight at 4 °C with specific primary antibodies: Sema3B (1:100, R&D Systems, USA), PlexinA1 (1:100, Abcam, USA), Iba1 (1:500, Abcam, USA), and NeuN (1:500, Abcam, USA). Subsequent to primary antibody incubation and PBST washes, appropriate fluorescent secondary antibodies (Invitrogen, 1:200) were applied for 2 h at room temperature. After final washes and DAPI counterstaining, imaging was performed on a Nikon confocal system.

### 2.16. Statistical Analysis

Data are presented as the mean ± standard error of the mean (SEM). All statistical analyses were performed using GraphPad Prism (v. 10.1). The normality of data distribution was verified with the Shapiro–Wilk test. For two-group comparisons, an unpaired, two-tailed Student’s *t*-test was applied. For comparisons among three or more groups, one-way analysis of variance (ANOVA) was utilized after ensuring homogeneity of variances with the Brown-Forsythe test. Post hoc testing was conducted with Bonferroni’s correction. Non-normally distributed data were analyzed using the Kruskal–Wallis test. A *p*-value of less than 0.05 was defined as the threshold for statistical significance.

## 3. Results

### 3.1. Decreased Sema3B in Perihematomal Tissue Is Associated with Neurological Recovery After ICH

**Figure 1:**
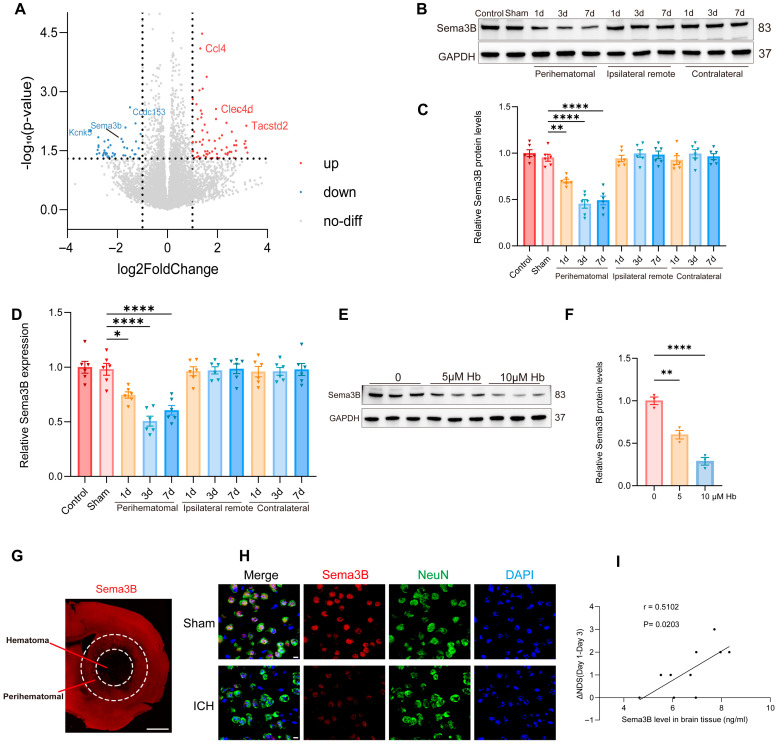
Decreased Sema3B in perihematomal tissue is associated with neurological recovery after ICH. (**A**) Volcano plot from RNA-seq comparing perihematomal tissue following ICH with Sham controls, showing differentially expressed genes; *Sema3b* is markedly downregulated. Abbreviations: *Sema3b*, semaphorin 3B; *Ccl4*, C–C motif chemokine ligand 4; *Clec4d*, C-type lectin domain family 4 member D; *Tacstd2*, tumor-associated calcium signal transducer 2; *Kcnk5*, potassium two pore domain channel subfamily K member 5; *Ccdc153*, coiled-coil domain containing 153. (**B**,**C**) Immunoblotting for relative Sema3B protein expression in perihematomal tissue from 0 h (Sham) to 7 days after ICH (*n* = 6). (**D**) Quantitative PCR assessment of relative Sema3B transcript abundance in perihematomal tissue from 0 h (Sham) to 7 days after ICH (*n* = 6). (**E**,**F**) Representative immunoblots showing relative Sema3B protein levels in N2a cells stimulated with hemoglobin (Hb) (*n* = 3). (**G**) Representative immunofluorescence images showing Sema3B expression in the perihematomal region at 72 h after ICH (*n* = 3; scale bar = 1 mm). (**H**) Representative confocal immunofluorescence images showing neuronal Sema3B expression at 0 h (Sham) and at the 72 h time point after ICH (*n* = 3; scale bar = 10 μm). (**I**) Correlation analysis revealed that higher Sema3B protein levels in the perihematomal tissue on day 3 after ICH were associated with a faster rate of neurological functional recovery during the first three days (*n* =10). (* *p* < 0.05, ** *p* < 0.01, **** *p* < 0.0001).

To uncover key molecular players in ICH pathogenesis, we profiled the transcriptomic profiles of perihematomal tissues from ICH models alongside Sham-operated controls using RNA sequencing (RNA-seq). A total of 114 genes were differentially expressed after ICH, among which Sema3B—relatively understudied within the Sema3 family—was prominently downregulated (Figure 1A). Western blotting revealed a progressive decrease in Sema3B in perihematomal tissue after ICH, showing the most pronounced reduction at the day-3 time point (Figure 1B,C). Consistent results were obtained at the mRNA level by qRT-PCR (Figure 1D). In addition, exposing N2a cells to hemoglobin (Hb; 0–10 μM) led to a dose-dependent reduction in Sema3B protein (Figure 1E,F). Representative immunofluorescence images showed reduced Sema3B expression in the perihematomal region at 72 h after ICH (Figure 1G). Co-immunofluorescence of NeuN and Sema3B further demonstrated decreased neuronal Sema3B expression at this time point (Figure 1H), consistent with the RNA-seq data. To investigate the potential association between decreased Sema3B and ICH, we performed neurological function scoring on mice post-ICH modeling and measured Sema3B concentration in the perihematomal tissue on day 3 using ELISA. Correlation analysis revealed that mice with higher levels of Sema3B protein in the perihematomal tissue on day 3 also exhibited faster neurological functional recovery over the first three days (Figure 1I).

### 3.2. Sema3B Facilitates Microglial Clearance of the Hematoma Through PlexinA1 After ICH

**Figure 2:**
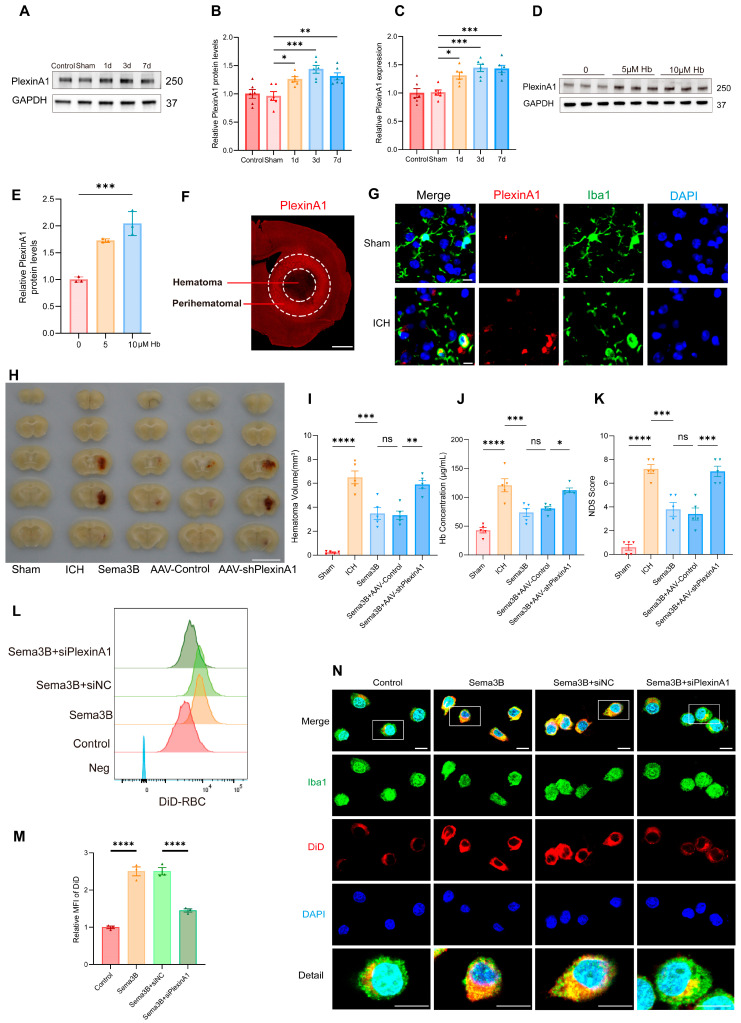
Sema3B facilitates microglial clearance of the hematoma through PlexinA1 after ICH. (**A**,**B**) Protein immunoblot analysis of relative PlexinA1 protein expression in perihematomal tissue from 0 h (Sham) to 7 days after ICH (*n* = 6). (**C**) qPCR measurement of relative PlexinA1 mRNA levels in perihematomal tissue from 0 h (Sham) to 7 days after ICH (*n* = 6). (**D**,**E**) Western blot detection of relative PlexinA1 protein abundance in microglia stimulated with Hb (*n* = 3). (**F**) Representative immunofluorescence images showing PlexinA1 expression in the perihematomal region at 72 h after ICH (*n* = 3; scale bar = 1 mm). (**G**) Representative confocal immunofluorescence images showing PlexinA1 expression in microglia at 0 h (Sham) and on day 3 following ICH (*n* = 3, scale bar = 10 μm). (**H**) Representative schematic of hematomas on day 3 after ICH (*n* = 5). (**I**) Quantification of hematoma volume on day 3 after ICH in each group (*n* = 5). (**J**) Analysis of residual hemoglobin content on day 3 after ICH (*n* = 5). (**K**) Neurological deficit scores (NDS) on day 3 after ICH (*n* = 5). (**L**,**M**) Representative flow-cytometry histograms and quantification of mean fluorescence intensity (MFI) for erythrophagocytosis in microglia under the indicated treatments (*n* = 3). (**N**) Representative confocal immunofluorescence images of erythrophagocytosis in microglia under the indicated treatments (*n* = 3, scale bar = 10 μm) (ns = no significance, * *p* < 0.05, ** *p* < 0.01, *** *p* < 0.001, **** *p* < 0.0001).

Our observation that higher Sema3B levels correlate with faster neurological recovery after ICH intrigued us, suggesting a potential role for Sema3B in promoting microglial phagocytosis of the hematoma. To further validate this hypothesis, we examined the expression changes of PlexinA1, the receptor for Sema3B, following ICH. Western Blot analysis revealed a time-dependent upregulation of PlexinA1 in mice after ICH compared to the sham group, peaking on day 3 (Figure 2A,B). Concurrently, quantitative real-time PCR (qPCR) results confirmed this trend at the mRNA level (Figure 2C). Furthermore, in microglia stimulated with hemoglobin (Hb), the protein level of PlexinA1 increased in a concentration-dependent manner with Hb (Figure 2D,E). Subsequently, immunofluorescence staining showed increased PlexinA1 expression in the perihematomal region at 72 h after ICH (Figure 2F). Co-immunofluorescence of Iba1 and PlexinA1 demonstrated increased PlexinA1 expression in microglia at day 3 after ICH (Figure 2G). Based on these data, we conclude that neuron–microglia interactions mediated by the Sema3B–PlexinA1 signaling pathway may be crucial in the pathophysiology of ICH.

To investigate how the altered Sema3B/PlexinA1 signaling after ICH influences the mechanisms of neurological recovery, we modulated this pathway in vivo prior to ICH induction using saline, Sema3B, and AAV-shPlexinA1 to achieve up- and downregulation. Assessment of hematoma volume on day 3 post-ICH indicated a marked reduction in mice receiving Sema3B alone compared to the vehicle controls. In contrast, the therapeutic effect of Sema3B was attenuated when administered in combination with AAV-shPlexinA1, resulting in a significantly larger residual hematoma (Figure 2H,I). Measurement of Hb content in the hemisphere affected by ICH revealed a reduction in the Sema3B-only group compared with vehicle, while mice receiving the combination of Sema3B and AAV-shPlexinA1 exhibited higher Hb content than those treated with Sema3B alone (Figure 2J). With respect to neurological recovery, mice treated with Sema3B alone had lower NDS scores (better function) on day 3 after ICH, which deteriorated upon AAV-shPlexinA1 administration (Figure 2K). In vitro, flow cytometry–based phagocytosis assays demonstrated that Sema3B markedly enhanced erythrophagocytosis by microglia, with increased mean fluorescence intensity (MFI); knockdown of PlexinA1 with siPlexinA1 reduced erythrophagocytic capacity (Figure 2L,M). Consistent findings were observed by immunofluorescence (Figure 2N). Collectively, these data indicate that Sema3B/PlexinA1 regulates microglial phagocytosis of hematoma and promotes neurological recovery.

### 3.3. Sema3B Increases HO-1 and TREM2 Expression in Mice and Microglia After ICH

**Figure 3:**
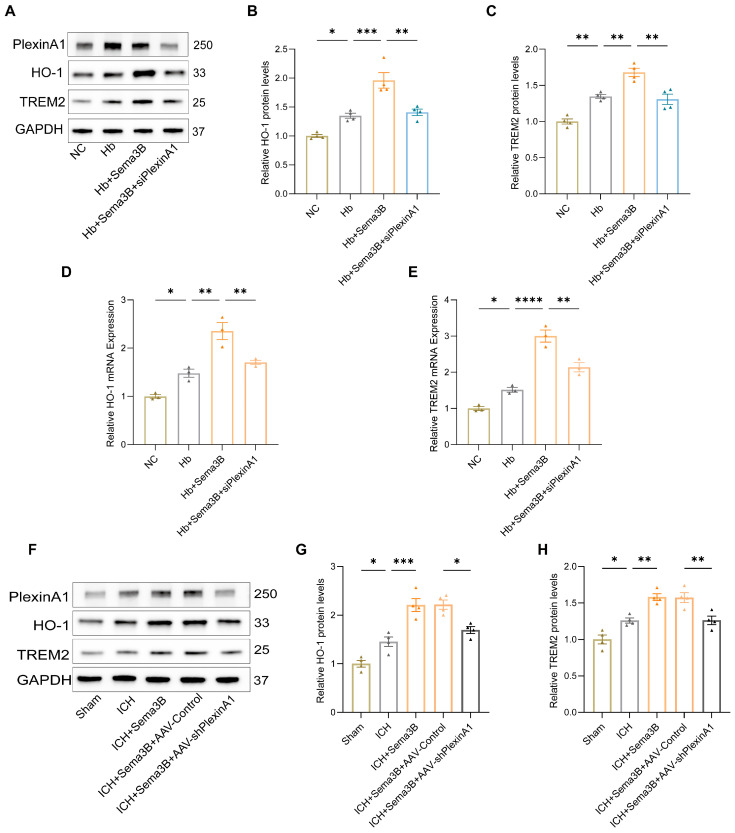
Sema3B increases HO-1 and TREM2 expression in perihematomal brain tissue and microglia after ICH. (**A**–**C**) Immunoblotting showing relative HO-1 and TREM2 protein expression in microglia under the different treatments (*n* = 4). (**D**,**E**) Quantitative PCR (qRT-PCR) assessment of HO-1 and TREM2 transcript levels in microglia under the different treatments (*n* = 3). (**F**–**H**) Protein analysis by Western blot for HO-1 and TREM2 in perihematomal tissue from ICH mice on post-ICH day 3 under the different treatments (*n* = 4) (* *p* < 0.05, ** *p* < 0.01, *** *p* < 0.001, **** *p* < 0.0001).

To investigate the mechanism by which the Sema3B/PlexinA1 pathway regulates microglial phagocytic capacity, we examined HO-1 and TREM2—key mediators of hematoma resolution and hemoglobin clearance that influence neurological recovery. Western blotting of microglia treated with Hb, Sema3B, and siPlexinA1 showed that Sema3B increased HO-1 and TREM2 protein expression, whereas PlexinA1 knockdown abrogated this effect (Figure 3A–C); qRT-PCR yielded concordant results (Figure 3D,E). In the ICH mouse model, perihematomal brain tissues from mice treated with Sema3B and AAV-shPlexinA1 exhibited similar changes in HO-1 and TREM2 expression (Figure 3F–H).

### 3.4. Sema3B Upregulates HO-1/TREM2 Expression via Nrf2

**Figure 4:**
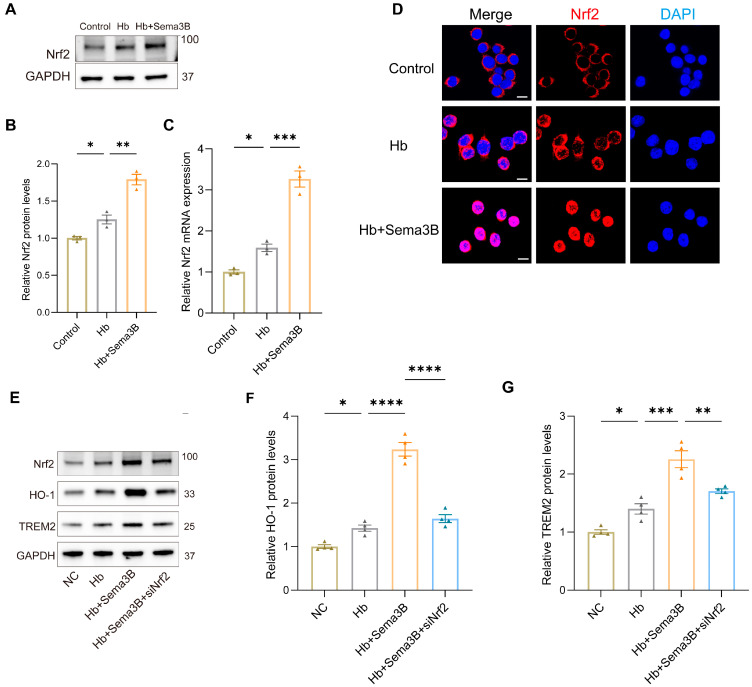
Sema3B upregulates HO-1 and TREM2 expression via Nrf2. (**A**,**B**) Immunoblotting for relative Nrf2 protein expression in microglia under the different treatments (*n* = 3). (**C**) Quantitative PCR (qRT-PCR) assessment of relative Nrf2 transcript levels in microglia under the different treatments (*n* = 3). (**D**) Confocal micrographs depicting Nrf2 expression in microglia under the different treatments (*n* = 3; scale bar = 10 μm). (**E**–**G**) Protein analysis by Western blot for HO-1 and TREM2 in microglia under the different treatments (*n* = 3) (* *p* < 0.05, ** *p* < 0.01, *** *p* < 0.001, **** *p* < 0.0001).

To further elucidate the mechanism by which Sema3B increases HO-1 and TREM2, we focused on Nrf2 based on its critical function in the transcriptional response to oxidative stress and its known contribution to hematoma resolution post-ICH. In a microglia in vitro ICH model, Western blotting and PCR showed that Sema3B treatment elevated Nrf2 protein and mRNA levels (Figure 4A–C). Confocal immunofluorescence of similarly treated microglia revealed a marked increase in total Nrf2 and nuclear translocation of Nrf2 in the Sema3B group (Figure 4D). Subsequently, microglia exposed to Hb and Sema3B were transfected with siNrf2; the Sema3B-induced upregulation of HO-1 and TREM2 was suppressed in siNrf2-treated cells (Figure 4E–G). These findings point to Nrf2-dependent upregulation of HO-1 and TREM2 as a key mechanism through which Sema3B facilitates hematoma clearance and improves neurological recovery after ICH.

### 3.5. Sema3B Enhances the Phagocytosis of Erythrocytes by Microglia via the TREM2/DAP12/SYK/PI3K/AKT Pathway

To further investigate the signaling mechanism by which Sema3B enhances microglial phagocytosis, we focused on the TREM2/DAP12/SYK/PI3K/AKT pathway, which is closely associated with hematoma clearance and neurological recovery after ICH. BV2 microglia were divided into the following groups: control, Hb, Sema3B, Hb + Sema3B, Hb + Sema3B + siNC, and Hb + Sema3B + siTREM2, followed by Western blot analysis of pathway-related proteins.

**Figure 5:**
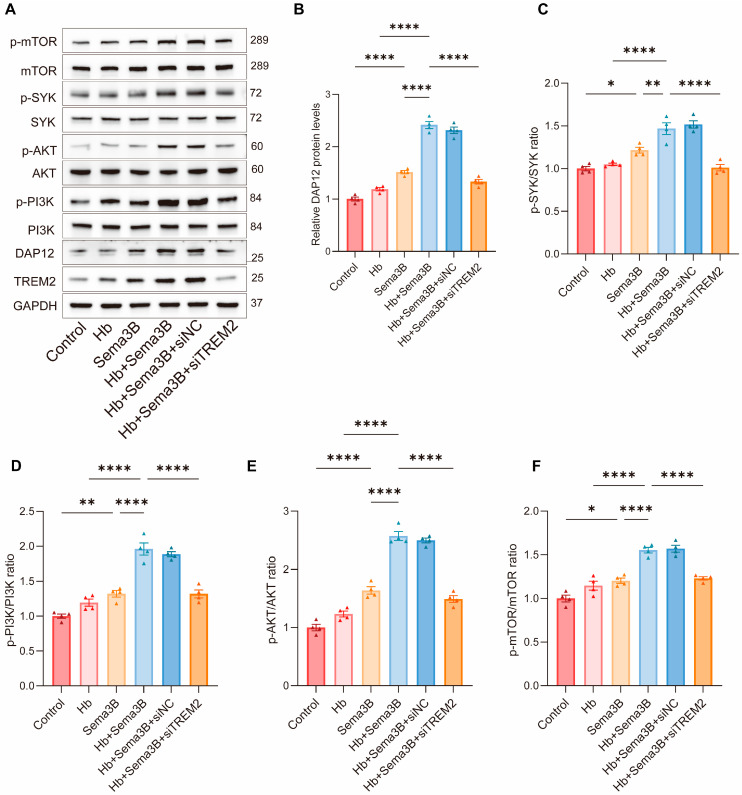

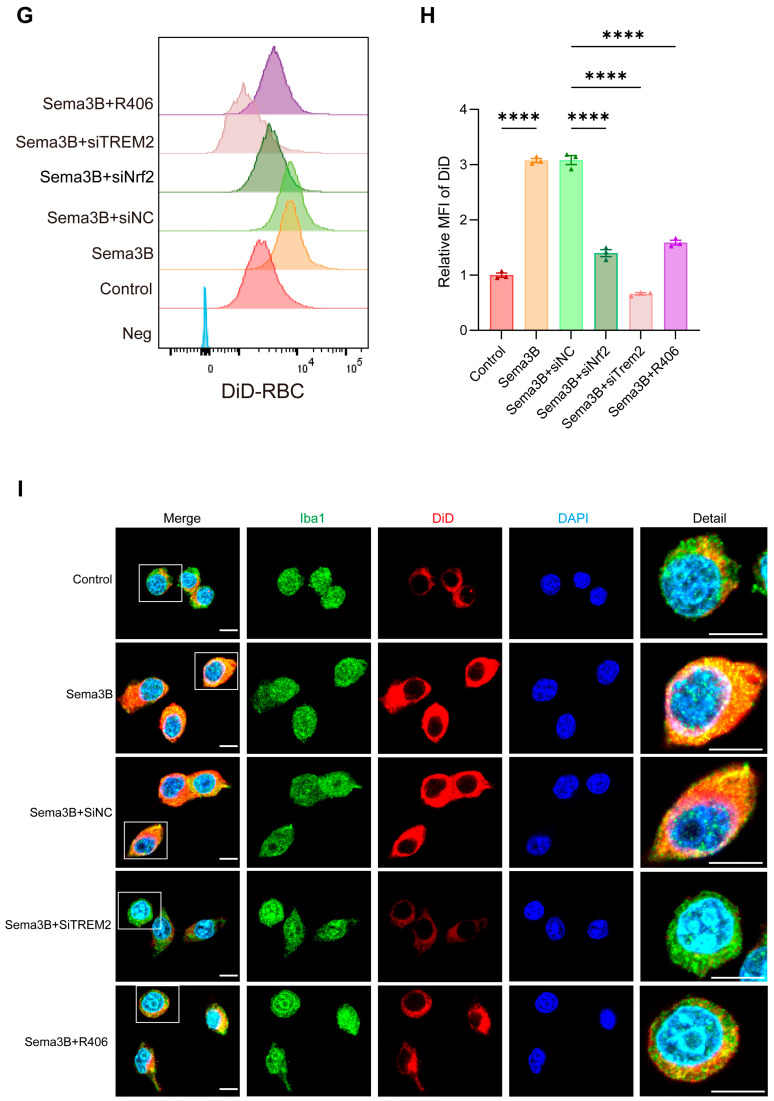
Sema3B enhances erythrophagocytosis by microglia via the TREM2/DAP12/SYK/PI3K/AKT pathway. (**A**–**F**) Immunoblotting for relative levels of DAP12, phospho-SYK, phospho-PI3K, phospho-AKT, and phospho-mTOR in microglia under the different treatments (*n* = 3). (**G**,**H**) Representative flow-cytometry histograms and quantitative analysis of the MFI for erythrophagocytosis in microglia under the different treatments (*n* = 3). (**I**) Confocal micrographs depicting erythrophagocytosis in microglia under the different treatments (*n* = 3, scale bar = 10 μm) (* *p* < 0.05, ** *p* < 0.01, **** *p* < 0.0001).

Treatment with Hb alone did not result in an apparent increase in the levels of DAP12, p-SYK, p-PI3K, p-AKT, or p-mTOR. In contrast, Sema3B treatment alone significantly increased the levels of DAP12 and the phosphorylation of SYK, PI3K, AKT, and mTOR. Notably, combined treatment with Hb and Sema3B led to a more pronounced elevation of these signaling proteins compared with Sema3B treatment alone, indicating activation of the TREM2/DAP12/SYK/PI3K/AKT pathway. Importantly, these effects were not altered by siNC but were markedly reversed by siTREM2, demonstrating that Sema3B-mediated activation of this pathway is dependent on TREM2 signaling (Figure 5A–F).

### 3.6. Interaction Between PlexinA1 and TREM2 in Microglia Is Enhanced by Sema3B, Synergistically Promoting Hematoma Phagocytosis

**Figure 6:**
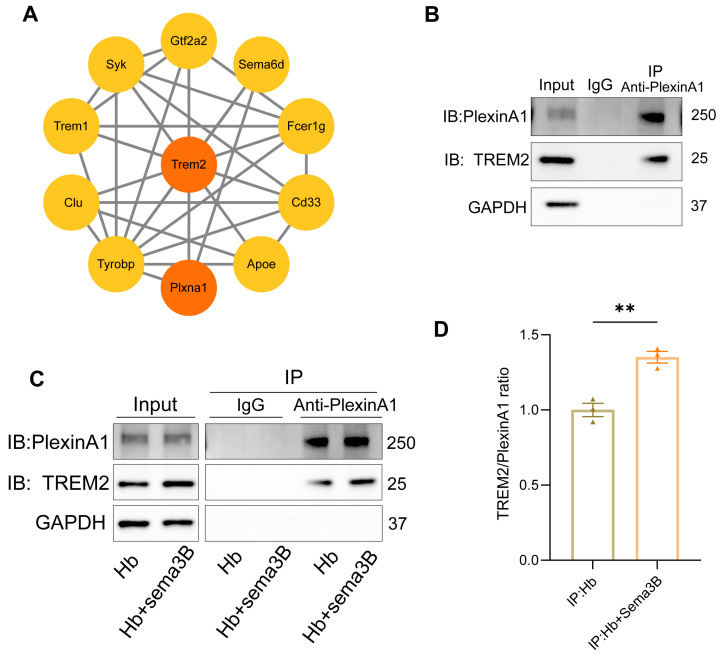
Interaction between PlexinA1 and TREM2 in microglia is enhanced by Sema3B, synergistically promoting hematoma phagocytosis. (**A**) The interaction network between PlexinA1 and TREM2 was determined using the STRING database (https://string-db.org (accessed on 2 June 2025)) and subsequently plotted with Cytoscape software (v3.10). (**B**) Detection of the PlexinA1–TREM2 interaction in microglia under basal conditions by co-immunoprecipitation and immunoblotting. (**C**,**D**) Co-immunoprecipitation and immunoblotting analyses of the PlexinA1–TREM2 interaction in microglia under the different treatments, with quantification (n = 3) (** *p* < 0.01).

Previous studies have reported an interaction between PlexinA1 and TREM2 [15]. Subsequently, we determined the interaction network between PlexinA1 and TREM2 using the STRING database and plotted it with Cytoscape software (v3.10) (Figure 6A). To determine whether a similar interaction exists in microglia and whether it is modulated by Sema3B in a manner conducive to microglial phagocytosis, co-immunoprecipitation (co-IP) was performed to assess PlexinA1–TREM2 binding in microglia. An interaction between PlexinA1 and TREM2 was detected in microglia (Figure 6B). Moreover, addition of Sema3B to microglia in the in vitro ICH model significantly increased the amount of TREM2 co-precipitating with PlexinA1 (Figure 6C,D), this indicates that Sema3B facilitates rapid, transcription-independent activation of the DAP12/SYK/AKT phagocytosis pathway by enhancing the interaction between PlexinA1 and TREM2. This mechanism ensures the immediate initiation and sustained amplification of microglial phagocytic function during the early stages of ICH, thereby synergizing with the conventional transcription factor-mediated upregulation of TREM2 to collectively promote hematoma clearance.

### 3.7. Sema3B Promotes Microglial Polarization Toward the M2 Phenotype and Ameliorates Microglia-Mediated Inflammation After ICH

**Figure 7:**
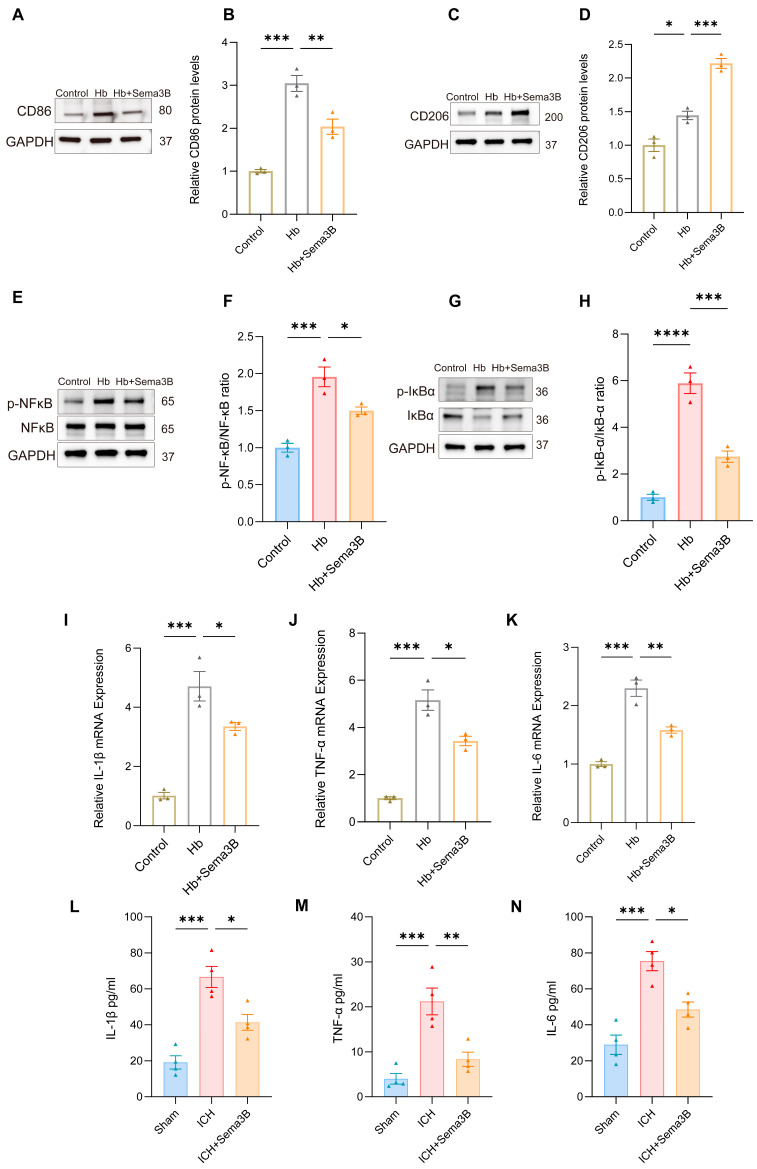
Sema3B promotes microglial polarization toward the M2 phenotype and ameliorates microglia-mediated inflammation after ICH. (**A**–**D**) Immunoblotting for relative protein expression of the M1 marker CD86 and the M2 marker CD206 in microglia under the different treatments (*n* = 3). (**E**–**H**) Protein analysis by Western blot for phosphorylated NF-κB and IκB-α in microglia under the different treatments (*n* = 3). (**I**–**K**) Quantitative PCR assessment of IL-1β, TNF-α, and IL-6 transcript levels in microglia under the different treatments (*n* = 3). (**L**–**N**) ELISA measurement of IL-1β, TNF-α, and IL-6 protein concentration in perihematomal tissue on post-ICH day 3 under the different treatments (* *p* < 0.05, ** *p* < 0.01, *** *p* < 0.001, **** *p* < 0.0001).

The rate of hematoma resorption and neurological recovery after ICH is tightly linked to microglial phenotypic shifts and the accompanying inflammatory response. To determine whether the beneficial effects of Sema3B on neurological recovery are related to modulation of microglial phenotype and inflammation, In a microglial ICH model, Sema3B shifted the balance from the M1 marker CD86 toward the M2 marker CD206 at the protein level (Figure 7A–D). This immunomodulatory profile was mirrored by decreased phosphorylation of NF-κB and IκB-α (Figure 7E–H). Furthermore, Sema3B significantly reduced the mRNA abundance of IL-1β, IL-6, and TNF-α in vitro (Figure 7I–K), a result that was consistent with reduced protein levels of these cytokines in perihematomal tissue at the 3-day time point following ICH (Figure 7L–N).

## 4. Discussion

**Figure 8:**
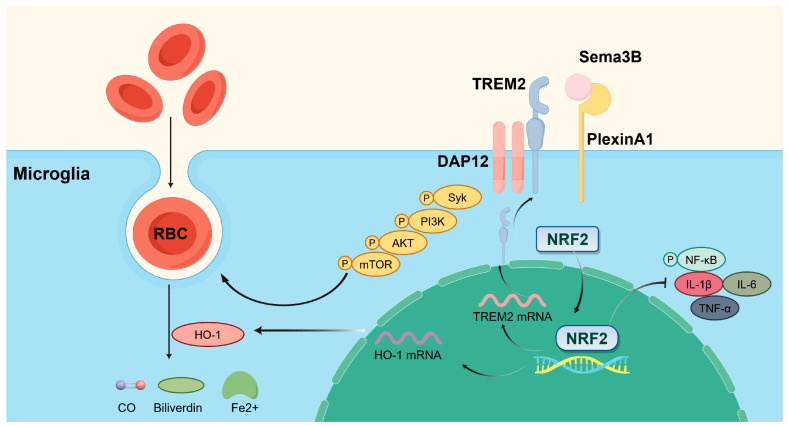
Sema3B promotes hematoma absorption after ICH by enhancing PlexinA1-mediated microglial phagocytic function. Exogenous supplementation of Sema3B binds to its receptor PlexinA1, activating the DAP12-dependent signaling pathway (Syk-PI3K-AKT-mTOR) and NRF2 in microglia, thereby increasing the expression of TREM2 and HO-1 to facilitate microglia-mediated hematoma clearance, while suppressing neuroinflammation by inhibiting the NF-κB pathway and reducing pro-inflammatory cytokines (IL-1β, IL-6, and TNF-α). This figure was drawn by Figdraw.

Collectively, our findings point to several key outcomes: (a) Sema3B expression was decreased and PlexinA1 expression was increased after ICH; (b) Sema3B enhanced the expression of HO-1 and TREM2 in microglia via Nrf2, thereby promoting microglial phagocytosis of hematoma and improving neurological function; (c) the ability of Sema3B to augment microglial erythrophagocytosis was primarily mediated through the TREM2/DAP12/SYK/AKT signaling pathway; (d) Sema3B promotes the PlexinA1-TREM2 interaction, enabling rapid activation and sustained amplification of the phagocytic pathway to synergistically enhance hematoma clearance; and (e) Sema3B promoted microglial polarization toward the M2 phenotype while suppressing ICH-induced microglial inflammation. The mechanisms of our study are shown in Figure 8.

The primary source of brain injury caused by ICH is the formation of the intraparenchymal hematoma. The mass effect and mechanical compression resulting from hematoma formation constitute the primary injury, leading to increased intracranial pressure, reduced cerebral perfusion, and brain herniation. Subsequently, erythrocyte lysis products released from the hematoma contribute to secondary injuries, including neurotoxicity, inflammation, and oxidative stress. Therefore, early hematoma clearance is critical for the treatment of ICH and for improving prognosis, underscoring the substantial therapeutic potential of strategies that promote endogenous hematoma resolution.

Our data provide the first evidence that ICH leads to a reduction in Sema3B expression, which is predominantly localized in neurons. This finding is consistent with observations in a mouse model of depression, where neuronal Sema3B expression was likewise downregulated [27]. The convergence of this phenomenon in two distinct neurological disorders—one acute (ICH) and one chronic (depression)—suggests that the loss of Sema3B may disrupt the crosstalk and reciprocal regulation between neurons and microglia, ultimately creating an environment prone to neuroinflammation and impaired repair. We also propose that the downregulation of Sema3B after ICH is potentially linked to the processes that underlie the onset and progression of post-ICH depression, an intriguing possibility warranting further investigation. In parallel, we observed an upregulation of PlexinA1 in microglia after ICH, consistent with previous reports of increased PlexinA1 expression in inflammation-induced activated microglia [29]. This upregulation of PlexinA1 on microglia may represent a “gain-of-function” mechanism, driving microglial inflammatory responses in the absence of the inhibitory signal provided by Sema3B.

The Nrf2/HO-1 pathway is widely regarded as a fundamental component of the body’s defense machinery, crucial for combating cellular damage induced by oxidative stress and inflammation after ICH. Its significance is well-documented in the context of protection from cytotoxic injury. Our finding that Sema3B enhances Nrf2 transcription in microglia raises the intriguing possibility of a broader role for semaphorins in regulating cellular stress pathways. Indeed, previous studies have reported that SEMA3A (another member of the Sema3 family) is transcriptionally regulated by NRF2 in human keratinocytes [30]. This potential crosstalk or feedback loop between semaphorins and the NRF2 axis in different cell types may represent an important area for future investigation. In agreement with earlier reports, we observed that Nrf2 regulates TREM2 protein expression in microglia [9]. Furthermore, our data demonstrated that Nrf2, HO-1, and TREM2 were all upregulated following ICH, consistent with findings from prior ICH studies [12,31]. While the increased expression of Nrf2, HO-1, and TREM2 after ICH may represent a physiological defense response, the critical observation from our study is that Sema3B significantly promoted their expression. The coordinated upregulation of Nrf2, HO-1, and TREM2 by Sema3B highlights a sophisticated cellular defense program: the antioxidant system (Nrf2/HO-1) alleviates oxidative damage, while the phagocytic receptor (TREM2) is simultaneously enhanced to facilitate debris clearance. This dual action may exert synergistic effects in limiting secondary injury and promoting repair after ICH. Looking forward, therapeutic strategies aimed at simultaneously activating Nrf2 and enhancing TREM2 represent a promising new direction for ICH research.

Subsequent investigation into how Sema3B boosts phagocytic function revealed a critical dependence on the TREM2/DAP12/SYK/PI3K/AKT pathway for the observed enhancement in microglial clearance of erythrocytes. TREM2/DAP12 plays a pivotal role in multiple CNS disorders by regulating microglial functions, including survival, phagocytosis, and cytokine production [32]. Moreover, TREM2-dependent phagocytosis is known to depend on the activation of the downstream DAP12/SYK/PI3K/AKT signaling cascade [33]. TREM2-mediated PI3K/AKT activation not only promotes phagocytosis but also improves neurological function after ICH and neuronal apoptosis and attenuates neuroinflammation [12]; recent work further indicates that TREM2-driven PI3K/AKT signaling alleviates white matter injury following ICH [13]. In our microglia ICH model, components of the TREM2/DAP12/SYK/PI3K/AKT/mTOR pathway—and/or their phosphorylation levels—were not significantly upregulated, and treatment with Sema3B produced a significant increase in these molecules and their phosphorylation compared with the vehicle group. Collectively, these observations suggest that the TREM2/PI3K/AKT pathway may represent a core mechanism governing neuroinflammation and reparative processes after ICH and merits continued investigation. Given that Sema3B is a molecule involved in axon guidance and synaptic plasticity, it remains to be determined whether exogenous supplementation directly contributes to the repair of injured neurons after ICH—thereby accelerating neurological recovery—beyond its effects on hematoma clearance and inflammation.

The physical interaction between PlexinA1 and TREM2, initially characterized in myeloid cells, participates in the modulation of immune responses and the maintenance of skeletal equilibrium [15]. Building on this foundational work, our study demonstrated that this interaction is also present in microglia. Furthermore, we confirmed that Sema3B facilitates a direct interaction between the PlexinA1 and TREM2 proteins within microglia. Binding of Sema3B may either induce conformational changes in PlexinA1 or facilitate its recruitment to pre-existing TREM2/DAP12-centered signaling hubs. This ligand-induced receptor complex formation enables highly efficient and direct crosstalk between Sema3B and the TREM2-mediated phagocytosis pathway, ensuring the immediate initiation and sustained amplification of microglial phagocytic function during the early stages of ICH. Therefore, our study not only confirms the long-term mechanism by which Sema3B upregulates TREM2 expression via the transcription factor Nrf2 and activates the DAP12/SYK/AKT pathway, but also unveils a novel, rapid, and transcription-independent pathway driven by Sema3B. This newly identified pathway involves Sema3B enhancing the direct interaction between PlexinA1 and pre-existing TREM2, thereby rapidly activating the phagocytosis pathway. This dual-mechanism model guarantees both the immediate launch and the subsequent sustained amplification of microglial phagocytic function in the acute phase of ICH, working in concert to promote hematoma clearance.

Following ICH, Nrf2 activation modulates the microglial polarization state, effectively shifting the balance from the detrimental M1 phenotype toward the beneficial M2 phenotype [34]. Moreover, Nrf2 activation reduces early brain injury after ICH through NF-κB inhibition, a mechanism already established in prior studies [35]. It is therefore logical that Sema3B drives a microglial phenotypic shift towards the anti-inflammatory M2 state and suppresses microglia-mediated inflammation after ICH, as confirmed by our findings. The anti-inflammatory effects of Sema3B may not be attributable solely to NF-κB suppression via Nrf2 activation but may also involve TREM2. Indeed, previous studies have shown that TREM2 overexpression ameliorates multiple pathological processes after ICH, including neurological impairment, apoptosis, neuroinflammation, and cerebral edema, through inhibition of NF-κB signaling [14].

Although our study provides convergent in vivo and in vitro evidence that Sema3B activates NRF2 and upregulates HO-1 and TREM2, thereby enhancing microglial hematoma phagocytosis, improving acute neurological outcomes, and attenuating inflammatory responses, several considerations warrant further investigation. First, our key assessments were focused primarily on the acute phase after ICH (with major readouts centered on post-ICH day 3), and thus the potential impact of Sema3B on subacute and chronic repair processes and long-term behavioral recovery remains to be defined with extended follow-up. Second, although we used the collagenase-induced ICH model in this study, future work may further evaluate the effects and underlying mechanisms of Sema3B in complementary models, including the autologous blood injection model, to enhance the generalizability of our findings. Third, the in vitro experiments largely relied on BV2 microglia stimulated with Hb, which is well suited for mechanistic validation; nevertheless, incorporating primary microglia and models that more closely reflect the in vivo perihematomal milieu would further strengthen the physiological relevance of our findings. Fourth, the intracerebroventricular delivery and intervention performed before ICH induction facilitated delineation of causality and early mechanisms; building on this framework, future work will evaluate therapeutic efficacy and safety across post-ICH dosing windows and dose ranges, and will explore delivery strategies with greater translational feasibility. Finally, given that Sema3B is a canonical axon-guidance cue with potentially broader biological actions, it will be important to determine whether additional downstream pathways or multicellular interactions contribute to neuronal repair, blood–brain barrier stability, and immune microenvironment remodeling after ICH, thereby informing the development of Sema3B-based combinatorial therapeutic strategies.

## 5. Conclusions

In summary, our study demonstrated that Sema3B promotes hematoma clearance, neurological recovery, and the alleviation of microglia-mediated inflammation after ICH through Nrf2 activation and the subsequent upregulation of its target genes, HO-1 and TREM2. Therefore, targeting Sema3B represents a promising therapeutic avenue to facilitate hematoma resolution following ICH.

## Data Availability

The original contributions presented in this study are included in the article. Further inquiries can be directed to the corresponding author.
